# Large-Scale Screening and Machine Learning for Metal–Organic Framework Membranes to Capture CO_2_ from Flue Gas

**DOI:** 10.3390/membranes12070700

**Published:** 2022-07-11

**Authors:** Yizhen Situ, Xueying Yuan, Xiangning Bai, Shuhua Li, Hong Liang, Xin Zhu, Bangfen Wang, Zhiwei Qiao

**Affiliations:** 1 Guangzhou Key Laboratory for New Energy and Green Catalysis, School of Chemistry and Chemical Engineering, Guangzhou University, Guangzhou 510006, China; 2112005038@e.gzhu.edu.cn (Y.S.); 2111905092@e.gzhu.edu.cn (X.Y.); baixiangning@e.gzhu.edu.cn (X.B.); lish@gzhu.edu.cn (S.L.); lhong@gzhu.edu.cn (H.L.); 2 Joint Institute of Guangzhou University & Institute of Corrosion Science and Technology, Guangzhou University, Guangzhou 510006, China

**Keywords:** membrane separation, metal–organic frameworks, machine learning

## Abstract

To combat global warming, as an energy-saving technology, membrane separation can be applied to capture CO_2_ from flue gas. Metal–organic frameworks (MOFs) with characteristics like high porosity have great potential as membrane materials for gas mixture separation. In this work, through a combination of grand canonical Monte Carlo and molecular dynamics simulations, the permeability of three gases (CO_2_, N_2_, and O_2_) was calculated and estimated in 6013 computation–ready experimental MOF membranes (CoRE–MOFMs). Then, the relationship between structural descriptors and permeance performance, and the importance of available permeance area to permeance performance of gas molecules with smaller kinetic diameters were found by univariate analysis. Furthermore, comparing the prediction accuracy of seven classification machine learning algorithms, XGBoost was selected to analyze the order of importance of six structural descriptors to permeance performance, through which the conclusion of the univariate analysis was demonstrated one more time. Finally, seven promising CoRE-MOFMs were selected, and their structural characteristics were analyzed. This work provides explicit directions and powerful guidelines to experimenters to accelerate the research on membrane separation for the purification of flue gas.

## 1. Introduction

With the rapid development of industry, global warming has increased, with increasing emissions of CO_2_, which is very harmful to the life and development of humans [1]. Because it is impossible to completely develop industry without carbon in a short time, the capture of CO_2_ has become an essential way to reduce greenhouse gas emissions and improve the present condition. However, CO_2_ capture is an energy-intensive process for a series of reasons, among which is the gas composition [1]. Therefore, it is an urgent need to develop energy-saving technology to separate CO_2_ from gas mixtures. Comparing with a series of separation technologies, membrane separation has received much attention due to its efficiency, low–energy consumption, and requirement of relatively simple equipment [2]. However, the search is on for a membrane that shows good separation performance with wide application, especially when complex components are involved. The development of materials with good membrane separation performance has become a hot topic in recent years.

In the last twenty years, new materials metal–organic frameworks (MOFs), self-assembled by a wide range of organic links and metal nodes, have been considered to have potential for use in domains such as drug delivery [3], catalysis [4], gas storage [5,6,7], gas adsorption and separation [8,9,10,11,12] due to their excellent characteristics such as large surface area and high porosity. Commonly, MOFs can be applied as adsorbates and membranes for the separation of gas mixtures. For example, designing porous materials at the molecular level for adsorption-based application, PCN–88 was synthesized by Li et al. [13] based on a new concept, ‘single-molecule trap’, which showed excellent preferential adsorption of CO_2_ over N_2_ and CH_4_. With two new empirical equations for the prediction of hydrogen adsorption capacity by pore volume by Zhang et al. [14], NPF–200 was predicted and demonstrated as a promising MOF for H_2_ storage. Further, an MOF, CAU–10–NH_2_, with excellent water stability/reusability was synthesized by their team [15], regarded as a promising material for C_2_H_2_/CO_2_ separation. Boyd et al. [16] synthesized two MOFs containing the most hydrophobic adsorbaphore found by the evaluation of the CO_2_/N_2_ selectivity of MOFs in wet flue gas through computational screening. They found that both of their CO_2_/N_2_ separation performances were not affected by water. Nugent et al. [17] demonstrated the feasibility of a crystal engineering or reticular chemistry strategy, which controls pore functionality and size for the improvement of CO_2_ separation performance, through the synthesis of SIFSIX–2–Cu, SIFSIX–2–Cu–i, and SIFSIX–3–Zn. Although adsorption technology can be used to separate gas mixtures, membrane separation is an attractive option for its lower energy consumption [2]. In the field of membrane separation, MOF membranes (MOFMs) have been demonstrated as a kind of material with great potential to separate gas mixtures [2]. For example, Yin et al. [18] synthesized a thin tubular CAU–1 membrane exhibiting a high permeance of up to 1.34 × 10^−6^ mol·m^−2^·s^−1^·Pa^−1^ for CO_2_ and excellent selectivity of 17.4–22.8 for CO_2_/N_2_ mixture. Chang et al. [19] found that with the coating of a Pebax^®^1657 layer on the surface, the H_2_/CO_2_ separation performance of a ZIF–7–NH_2_ membrane can be improved. Kang et al. [20] found that the 1,2–bi–(4–pyridyl) ethylene (BPE) molecule distributed in channels can improve the H_2_/CO_2_ separation performance of the [Ni_2_(_L_–asp)_2_(BPE)]·(G) membrane. Under a two-step coating process, a new MOF-based membrane (PAN–*γ*–CD–MOF–PU membrane) was fabricated by Fan et al. [21], with a permeability to CO_2_ of over 70 barrer and selectivity to CO_2_/N_2_ and CO_2_/O_2_ of 253.46 and 154.28, respectively. Yan et al. [22] synthesized a UiO–66 membrane through tertiary growth at room temperature, which exhibited an optimal selectivity of 37.8 for CO_2_/N_2_. Chen et al. [23] fabricated a ZIF–8 membrane under different reaction conditions, and found that the optimal temperature for synthesis of ZIF–8 membrane is 80 °C. Further, they also found that the separation factor of CO_2_/N_2_ was 5.49 and the permeance of CO_2_ was 0.47 × 10^–7^ mol·m^–2^·s^–1^·Pa^–1^ under optimal conditions. However, with the increasing number of MOFs synthesized by experiments and built by computer technology, it is not practical to select potential MOFMs only through experiments due to the high costs and long time periods involved. Further, a series of chemical reagents are harmful to experimenters and the environment. There is an urgent need to develop a rapid method to select MOFMs with excellent performance.

Recently, high-throughput computational screening based on molecular simulation technology has been demonstrated as a useful way to accelerate the research on MOFMs by previous studies. For example, Qiao et al. [24] calculated the performance of 137,953 MOFMs by grand canonical Monte Carlo (GCMC) and molecular dynamics (MD) simulations and finally screened 24 optimal MOFMs for CO_2_/N_2_/CH_4_ separation. Appling GCMC and equilibrium MD simulations, Glover et al. [25] studied the separation performance of MOFMs for CO_2_/CH_4_ and H_2_S/CH_4_. They screened eight top-performing MOFMs superior than polymer membranes, zeolite membranes, and mixed matrix membranes (MMMs). Azar et al. [26] analyzed the H_2_/N_2_ separation performance of more than 3000 different types of MOF membranes and examined their separation potential in MMMs by molecular simulation. They found the characteristics of most promising MOFMs and the great advantage of incorporating MOFs into polymers. In the same way, Daglar et al. [27] screened optimal membranes for CO_2_/N_2_/H_2_O separation and explored the structure–performance relationship. They found that MOFMs with narrow pores, low surface areas, and monoclinic and lanthanide-containing structures are the best candidates for CO_2_/N_2_ membrane separation. Altintas et al. [28] used molecular simulation to explore the relationship between 175 different structures of MOFMs and the separation performance of C_2_H_6_/C_2_H_4_ and C_2_H_6_/CH_4_. They found that MOFMs with high C_2_H_6_ selectivity are those with cavity diameters between 6 and 9 Å, porosities lower than 0.5 and surface areas between 500 and 1000 m^2^g^−1^. Wang et al. [29] studied H_2_/CH_4_ separation using an IRMOF-1 membrane through a dual-force zone nonequilibrium molecular dynamics simulation. They reached the conclusion that both structural and chemical features of functionalized MOFMs determine gas separation performance. Bai et al. [30] screened MOFMs for the separation of gas pairs including H_2_ by molecular simulation and machine learning, and found 15 MOFMs with excellent separation performance.

Commonly, there are several compositions of industrial flue gas in addition to CO_2_, such as N_2_ and O_2_ [31,32,33]. In this work, the volume ratio of CO_2_, N_2_, and O_2_ is considered as 1:1:1. For the screening of MOFMs with great potential for ternary CO_2_/N_2_/O_2_ separation, the permeability (*P*) of pure CO_2_, N_2_, and O_2_ in MOFMs was calculated by GCMC and MD simulations. Then, through univariate analysis and machine learning, the relationship between structural descriptors and permeance performance was explored. Finally, seven promising MOFMs for ternary gas pair separation were screened.

## 2. Methods

### 2.1. Model

MOFMs studied in this work were MOFs from the 2019 computation–ready experimental metal–organic frameworks (CoRE–MOFs) database [34]. The structural parameters of CoRE–MOFs were derived from experimental data after free solvent molecules were removed [34,35]. The atomic structure of MOFs was described by Lennard–Jones (LJ) parameters and electrostatic potentials

(1) $$u_{LJ+\mathrm{elec}}\left(r\right)=\sum 4\varepsilon_{\mathrm{ij}}[{\left(\frac{\sigma_{ij}}{r_{ij}}\right)}^{12}-{\left(\frac{\sigma_{ij}}{r_{ij}}\right)}^{6}]+\sum \frac{q_{i}q_{j}}{4\pi \varepsilon_{0}r_{\mathrm{ij}}}$$

where *ε_ij_* is the potential energy parameter, *σ_ij_* represents the equilibrium distance between atoms, and *r_ij_* is the distance between atom pairs. *q_i_*, *q_j_* is the atomic charge of atoms *i* and atoms *j*, *ε*_0_ = 8.8542 × 10^−12^ C^2^∙N^−1^, which represents the permittivity of vacuum. The atomic charge of MOFs was quickly calculated by the MEPO-Qeq method [36]. All LJ potential energy parameters of MOFs are listed in Table S1, and they come from universal force field (UFF) [37]. The structural characteristics of MOFs are represented by six descriptors, in which the volumetric surface area (VSA) and fraction (*ϕ*) were calculated using N_2_ with a diameter of 3.64 Å and He with a diameter of 2.58 Å as a probe using the RASPA software package, and both the pore limited diameter (PLD) and largest cavity diameter (LCD) were estimated by the Zeo++ software package [38,39].

For CO_2_, O_2_, and N_2_ molecules, force field parameters were adopted from the transferable potentials for phase equilibria (TraPPE) force field [40], listed in Table S2. For CO_2_, the bond length of C–O is 1.16 Å, and the bond angle ∠OCO is 180°. For N_2_, which is considered as a three-site model, the N–N bond length is 1.10 Å. O_2_ is a three–site atom. A large number of studies have demonstrated that the application of UFF for MOFs and TraPPE for gases can accurately predict the gas adsorption and diffusion in various MOFs [41,42,43,44].

### 2.2. Molecular Simulation

In this work, the adsorption, diffusion, and permeability behaviors of pure CO_2_, O_2_, and N_2_ in MOFMs were simulated by GCMC and MD. Each GCMC or MD simulation was independently carried out and the interaction between MOFs and adsorbate molecules was calculated by the Lorentz–Berthelot rule. The periodic boundaries were applied in the three–dimensional system and the unit cell of each MOF was expanded to at least 24 Å in all the dimensions. To calculate LJ interactions, the spherical cutoff was set to 12 Å for long-range correction, and the framework–gas and gas–gas electrostatic interactions were calculated by Ewald summation [45]. Each GCMC simulation was run for 10,000 cycles, with the first 5000 cycles for the equilibration of simulation system and the last 5000 cycles for ensemble averages. Each cycle consisted of *n* trial moves (*n*: the number of adsorbate molecules), including translation, rotation, regrowth, and swap (insertion and deletion). The final simulation state of GCMC was used as the initial simulation state of MD. The MD duration in each MOF was 7 ns with the last 5 ns for production. All simulations of GCMC and MD were run under the RASPA software package [38]. After GCMC and MD simulations of each MOFM, the permeability of pure gases was calculated by

*P_i_ = K_i_ × D_i_* (2)

where *D_i_* is the diffusivity of component *i* in MOFMs, and *K_i_* is Henry’s constant of component *i*.

### 2.3. Evaluation of the Performance of MOFMs 

Normally, evaluation of the performance of MOFMs includes gas permeance performance and gas diffusion performance. To select a series of MOFMs to separate ternary gas pairs, the separation performance of MOFMs for two binary gas pairs was firstly analyzed considering the complexity of ternary gas pairs. In this work, considering the adsorption–diffusion mechanism in the process of porous membrane separation, adsorption selectivity (*S_ads_*) has also been considered, which was calculated by

*S_ads (i/j)_ = K_i/_K_j_* (3)

Meanwhile, gas diffusion performance was evaluated by the *D_i_* and diffusion selectivity (*S_diff_*), where *S_diff_* was calculated by

*S_diff (i/j)_ = D_i/_D_j_* (4)

and gas permeance performance was evaluated by *P_i_* and permselectivity (*S_perm_*), where *S_perm_* was calculated by

*S_perm (i/j)_ = P_i_/P_j_ = S_diff(i/j)_ S_ads (i/j)_* (5)

### 2.4. Machine Learning

To comprehensively analyze the relationship between structural descriptors and the permeance performance of MOFMs, seven classification machine learning algorithms were used to predict categories of MOFMs and calculate the relative importance (RI) of structural descriptors, which are support vector machine (SVM), *k*-nearest neighbor (KNN), decision tree (DT), random forest (RF), gradient boosting decision tree (GBDT), light gradient boosting machine (LGBM), and extreme gradient boosting (XGBoost).

At this stage, two categories were divided from the middle based on the permeance performance of MOFMs. Further, six structural descriptors (LCD, PLD, VSA, *ϕ*, the density (*ρ*), and the pore size distribution percentage between 2.5 and 3.5 Å (PSD%_(2.5–3.5)_)) were applied, in which PSD%_(2.5–3.5)_ was calculated by

PSD%_(*d*1−*d*2)_ = A_12_/A_total_ × 100% (6)

where A_total_ is the area under the entire PSD curve for a given MOF and A_12_ is the area between two pore sizes, *d*_1_ and *d*_2_. With a larger PSD%, there is a more significant proportion of uniform pores.

With five permeance performances of three components, in order to obtain a uniform calculation method for RI, after simply comparing the prediction accuracy of the algorithm, the optimal algorithm was selected. Then, after comparing the accuracy and stability of the optimal algorithm in detail with *k*-fold cross validation (*k* = 5, 10, and 15) five times, the best model with the best number fold cross validation was selected to calculate the RI of structural descriptors. In our work, the prediction accuracy of the machine learning model is evaluated by the accuracy (A), the sensitive (SEN), and the specificity (SPC). More detailed descriptions of the above algorithms are presented as Supplementary Materials.

## 3. Results and Discussion

### 3.1. Univariate Analysis

**Figure 1:**
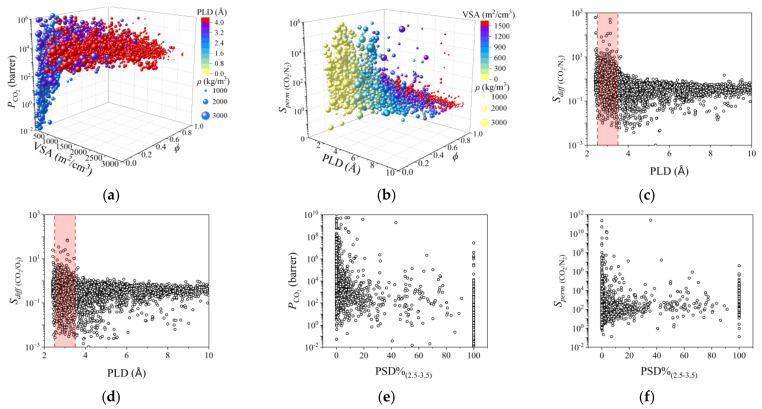
Structures–performance relationship studied by univariate analysis. (**a**) *P*_CO_2__–VSA, *ϕ*, PLD, and *ρ*; (**b**) *S_perm_* _(CO_2_/N_2_)_–VSA, *ϕ*, PLD, and *ρ*; (**c**) *S_diff_*_(CO_2_/N_2_)_–PLD; (**d**) *S_diff_*_(CO_2_/O_2_)_–PLD; (**e**) *P*_CO_2__–PSD%_(2.5_–_3.5)_; (**f**) *S_perm_*_(CO_2_/N_2_)_ –PSD%_(2.5_–_3.5)_. In (**a**), the colors of balls represent PLD and the sizes of ball represent *ρ* of MOFMs. In (**b**), the colors of balls represent the VSA and the sizes of ball represent *ρ* of MOFMs.

**Figure 2:**
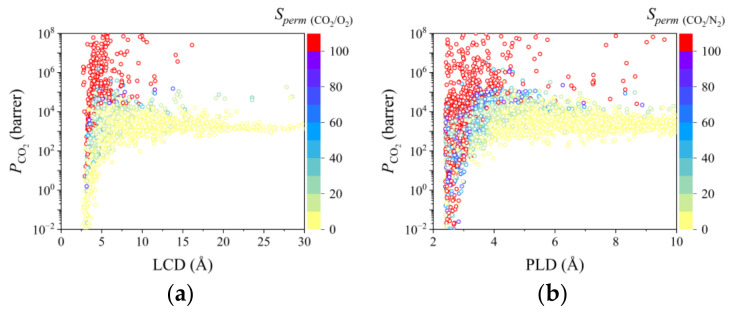
Relationship between LCD/PLD and permeance performance. (**a**) LCD–*P*_CO_2__–*S_perm_* _(CO_2_/O_2_)_; (**b**) PLD–*P*_CO_2__–*S_perm_* _(CO_2_/N_2___)_.

To efficiently screen top-performing MOFMs to separate ternary gas pairs, CO_2_/N_2_/O_2_, the relationship between the structural descriptors and permeance performance was analyzed. In Figure 1a and Figure 2a, with the increase in VSA, PLD, LCD, and *ϕ*, *P*_CO_2__ increased rapidly when they were low and finally plateaued. However, the relationship between *ρ* and *P*_CO_2__ was contrary. With a larger *ρ*, *P*_CO_2__ become smaller. A similar tendency can also be found for N_2_ and O_2_ in Figures S1a,b and S2c,d. In Figure 1b, Figure 2a,b, Figures S1c and S2a,b, for *S_perm_* _(CO_2_/O_2_)_ and *S_perm_* _(CO_2_/N_2_)_, most of them were both large when VSA, PLD, LCD, and *ϕ* were small, which decreased dramatically at first and then plateaued with the increase in VSA, PLD, LCD, and *ϕ*. The above phenomenon can be attributed to the size of available permeance area. Usually, gas molecules with smaller kinetic diameters can permeate through MOFMs even if the pore size of MOFMs is small. However, with a larger pore size, gas molecules with larger kinetic diameters can also spread through MOFMs if the pore size is large enough. For VSA, with a larger VSA, the available permeance area for gas molecules becomes larger. This indicates that the permeance performance of gas molecules with smaller kinetic diameters places a greater demand on the channel and geometry area of MOFMs.

Normally, the permeance performance of MOFMs is comprehensively decided by their adsorption and diffusion performance. Specially, diffusion performance is significantly related to PLD of MOFMs. So the relationship between diffusion performance and PLD was studied. In Figure S2e, *D*_N_2__ and *D*_O_2__ are similar, which is attributed to their similar kinetic diameter. Surprisingly, for CO_2_, with the smallest kinetic diameter in three gas molecules, *D*_CO_2__ is smaller than *D*_N_2__ and *D*_O_2__. This is because there are not only kinetic diameters but also other gas molecule properties impacting gas molecule diffusion. A similar phenomenon has also been found in previous research [24]. In Figure S2e, with the increase in PLD, *D* increases at first and then plateaus when PLD is larger than approximately 4 Å. For *S_diff_*, with a larger PLD, *S_diff_* shows a similar trend with *S_perm_*. For two binary gas pairs, PLD ranges from approximately 2.5 to 3.5 Å, and *P*_CO_2__ and *S_diff_* are both the largest. For better analysis of the relationship between structure and permeance performance, PSD%_(2.5–3.5)_ was used for the further study. However, a decreasing trend was found in Figure 1e,f and Figure S3.

**Figure 3:**
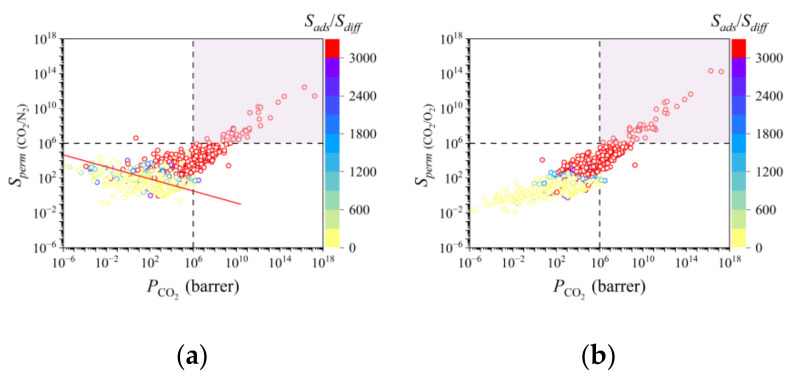
Relationship between *P*, *S_perm_*, and *S_ads_*/*S_diff_*. (**a**) *P_CO_2__*–*S_perm_*_(CO_2_/N_2_)_–*S_ads_*_(CO_2_/N_2_)_/*S_diff_*_(CO_2_/N_2_)_*;* (**b**) *P*_CO_2__–*S_perm_* _(CO_2_/O_2_)_–*S_ads_*_(CO_2_/O_2_)_/*S_diff_*_(CO_2_/O_2_)_. In (**a**), the red line represents the 2008 Robeson upper bound.

To better understand the separation mechanism, the relationship between *P*, *S_perm_*, and *S_ads_*/*S_diff_* was analyzed. In Figure 3a, the permeance performance of a large number of MOFMs for CO_2_/N_2_ separation was found to exceed the 2008 Robeson upper bound [46]. Further, it is easy to find that with the increase in *P*_CO_2__, *S_perm_* _(CO_2_/O_2_)_ increases, as shown in Figure 3b. The above phenomenon indicates that there is a great possibility of finding a series of MOFMs for ternary gas pair separation. Moreover, the *S_ads_*/*S_diff_* becomes larger when *P*_CO_2__ and *S_perm_* increase, which indicates that it is the adsorption mechanism and not the diffusion mechanism that plays a dominant role in membrane separation for CO_2_/N_2_ and CO_2_/O_2_ separation. This is attributed to the large quadrupole moment of CO_2_ impacting adsorption performance, which has been demonstrated previously [47,48,49,50].

### 3.2. Machine Learning

To comprehensively understand the order of impact importance of structural descriptors to the permeance performance of MOFMs, seven ML classification algorithms (SVM, KNN, DT, RF, GBDT, LGBM, and XGBoost) were applied to predict categories of MOFMs. Based on the performance of MOFMs, two categories were divided from the middle at first, in which P_1_ represents MOFMs with worse performance and P_2_ represents MOFMs with better performance. After comparison, XGBoost with optimal prediction performance was selected to predict categories of permeance performance.

**Figure 4:**
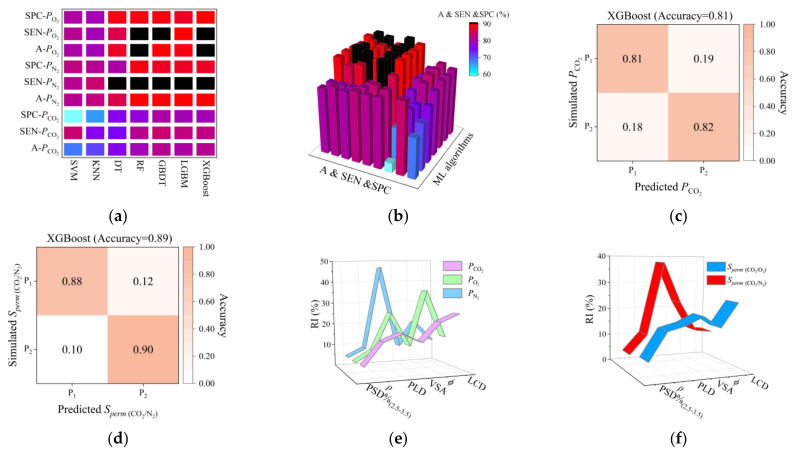
(**a**,**b**) Prediction accuracy comparison of seven classification algorithms; (**c**) confusion matrix for *P*_CO_2__; (**d**) confusion matrix for *S_perm_*_(CO_2_/N_2_)_; (**e**) RI comparison of *P*_CO_2__, *P*_O_2__, and *P*_N_2__; (**f**) RI comparison of *S_pe_*_rm (CO_2_/N_2_)_ and *S_perm_*_(CO_2_/O_2_)_.

From ML research, a series of conclusions were reached. (1) For *P*_CO_2__, XGBoost with 10-fold cross validation showed the best prediction. In Figure 4c, there was an accuracy of 81% in general, and an accuracy of 81% for P_1_ and 82% for P_2_ in the confusion matrix. According to ML calculation, the order of RI is LCD > *ϕ >* PLD > *ρ* > VSA > PSD%_(2.5–3.5)_. (2) For *P*_O_2__ and *P*_N_2__, the best prediction was by XGBoost with 15-fold cross validation. In Figure S14a,b, there was an accuracy of 90% in general for *P*_O_2__ and there was an accuracy of 91% in general for *P*_N_2__. In Figure 4e, the order of RI for *P*_O_2__ and *P*_N_2__ is similar—the order for *P*_O_2__ is *ϕ* > PLD > LCD >VSA > *ρ* > PSD%_(2.5–3.5)_ and for *P*_N_2__ is PLD > *ϕ* > LCD >VSA > *ρ* > PSD%_(2.5–3.5)_. (3) For *S_perm_* _(CO_2_/O_2_)_ and *S_perm_* _(CO_2_/N_2_)_, optimal prediction was by XGBoost with 10-fold and 5-fold cross validation. From ML calculation, the order of RI is shown in Figure 4f. For *S_perm_* _(CO_2_/O_2_)_, the order of RI is LCD > VSA > PLD > *ϕ* > *ρ* > PSD%_(2.5–3.5)_; while, for *S_perm_* _(CO_2_/N_2_)_, the order of RI is PLD > VSA > *ϕ* > *ρ* > LCD > PSD%_(2.5–3.5)_.

Based on the above conclusions from ML, we found that of six structural descriptors, LCD, PLD, and *ϕ* have a greater impact on gas molecule permeability. Further, the order of RI for LCD, VSA, and *ρ* for the permeability of three gas molecules is contrary to the order of RI of kinetic diameter. For *S_perm_*, the order of RI of LCD and *ρ* for *S_perm_* _(CO_2_/O_2_)_ is larger than *S_perm_* _(CO_2_/N_2_)_. Both confirm the conclusion by the univariate analysis that the permeance performance of gas molecules with smaller kinetic diameters places a greater demand on the channel and geometry area of MOFMs. On the contrary, the order of RI of PLD for the permeability of three gas molecules is the same as the order of RI of kinetic diameter (*Dia*), *Dia*_N_2__ > *Dia*_O_2__ > *Dia*_CO_2__, because PLD plays an important role in the confirmation of diffusion barrier and determines the gas molecule diffusion barrier in porous materials [51,52]. This is also explained by the phenomenon that the importance of PLD to *S_perm_* _(CO_2_/N_2_)_ is larger than *S_perm_* _(CO_2_/O_2_)_.

To better apply the ML result, the relationships between LCD and *S_perm_* _(CO_2_/O_2_)_ and between PLD and *S_perm_* _(CO_2_/N_2_)_ were analyzed in detail. From Figure 2a,b, the optimal LCD range for MOFMs applied to separate CO_2_/O_2_ gas pair mainly is approximately 2.5–7.5 Å and the optimal PLD range for MOFMs applied to separate CO_2_/N_2_ gas pair is approximately 2–5 Å.

### 3.3. Separation of CO_2_/N_2_/O_2_ Pairs

**Figure 5:**
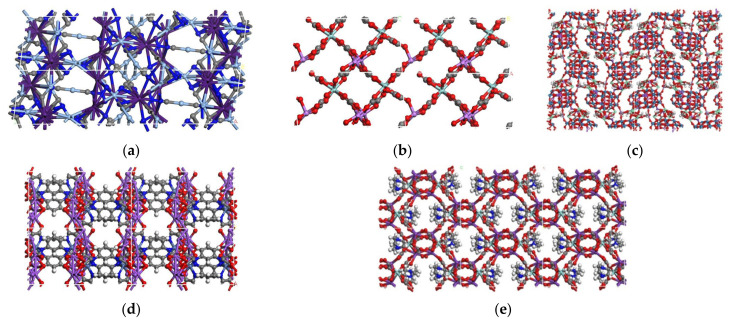
Atomistic structures of top-performing MOFMs. (**a**) CARGEI; (**b**) YUJWAD; (**c**) RIPWEU; (**d**) VEHNED; (**e**) WOCJII.

In this work, the vol% of CO_2_/N_2_/O_2_ pairs at 298 K and 1 bar is 1:1:1. For the purpose of CO_2_ capture instead of O_2_ and N_2_, it is essential use MOFMs with a large *P*_CO_2__, a small *P*_N_2__ and a small *P*_O_2__ even under large permselectivity. As such, 44 MOFMs used to separate CO_2_/O_2_ and 38 MOFMs used to separate CO_2_/N_2_ were, respectively, screened under the condition of *P*_CO_2__ ≥ 10^6^ barrer and *S_perm_* ≥ 10^6^ at first, as shown in the red area of Figure 3a,b. Of the above MOFMs, there are approximately 91% LCD of MOFMs for CO_2_/O_2_ separation under the optimal LCD range and approximately 84% PLD of MOFMs for CO_2_/N_2_ separation under the optimal PLD range, which demonstrates the effectiveness of ML analysis. Furthermore, in the search for promising MOFMs for practical application, seven top-performing MOFMs with *P*_N_2__ and *P*_O_2__ less than 100 barrer were selected, with structural characteristics found to completely follow the optimal LCD and PLD range. The details of seven top-performing MOFMs are listed in Table S10. Due to the similar atomistic structures of three MOFMs, the seven top-performing MOFMs are shown divided in Figure 5 and Figure S15.

Moreover, the ranges of other structural descriptors were analyzed. The VSA of the majority of MOFMs is smaller than 140 m^2^/cm^3^ and approximately 60% have a VSA smaller than 30 m^2^/cm^3^. The *ϕ* of the MOFMs is not greater than 0.3. The *ρ* of 71% of MOFMS is not larger than 1600 kg/m^3^. Further, the PSD%_(2.5–3.5)_ of 86% of MOFMs is less than 4%. The above conclusions on the range of optimal potential MOFMs once more demonstrates the importance of available permeance area for the permeance performance of gas molecules with smaller kinetic diameters.

## 4. Conclusions

In this work, the permeability of pure CO_2_, N_2_, and O_2_ in CoRE-MOFMs was calculated by GCMC and MD simulations to select MOFMs for the purification of flue gas. Univariate analysis showed the great impact of available permeance area to the permeance performance of gas molecules with smaller kinetic diameters. Further, the adsorption mechanism has a dominant role in the membrane separation mechanism for both CO_2_/N_2_ and CO_2_/O_2_ gas pairs. Furthermore, to comprehensively understand the order of importance of structural descriptors to permeance performance, seven classification algorithms were applied to predict categories of permeance performance, from which XGBoost was selected due to optimal prediction accuracy. Through ML calculations, the impact of available permeance area was demonstrated one more time. LCD and PLD were found to significantly impact the separation of CO_2_/O_2_ and CO_2_/N_2_, respectively. Finally, considering the purpose of CO_2_ capture, seven promising MOFMs with optimal permeance performance were screened. Their LCD and PLD completely conformed to the optimal LCD and PLD ranges by mining big data and ML, respectively. After the analysis of other structural descriptor ranges, the importance of available permeance area to permeance performance was illustrated for membrane separation by MOFMs. This work can provide explicit directions and powerful guidelines to study the capture of CO_2_ in flue gas by membrane separation.

## Data Availability

Data is contained within the article or Supplementary Material.

## Supplementary Materials

The following supporting information can be downloaded at: https://www.mdpi.com/article/10.3390/membranes12070700/s1, Figure S1: *P*_N_2__/*P*_O_2__/*S_perm_*_(CO_2_/O_2_)_**-**PLD, *φ*, VSA, and *ρ*; Figure S2: (a) LCD*-P*_CO_2__-*S_perm_* _(CO_2_/N_2_)_; (b) PLD*-P*_CO_2__-*S_perm_*_(CO_2_/O_2_)_; (c) *P*_N_2__-LCD; (d) *P*_O_2__-LCD; (e) *D*-PLD; Figure S3: *P*/*S_perm_*-PSD%_(2.5–3.5)_. Figure S4: The calculation schematic diagram for the accuracy, sensitive, and specificity; Figure S5: The accuracy, sensitive, and specificity comparison of seven algorithms fo*r S_perm_*_(CO_2_/N_2_)_ and *S_perm_*_(CO_2_/O_2_)_; Figure S6: The diagram of *k*-fold cross validation; Figure S7: KNN algorithm model; Figure S8: SVM algorithm model; Figure S9: DT algorithm model; Figure S10: RF algorithm model; Figure S11: GBDT algorithm model; Figure S12: The leaf-wise tree growth schematic diagram of LGBM algorithm model; Figure S13: XGBoost algorithm model; Figure S14: Confusion matrix from best model; Figure S15: Atomistic structures of top-performance MOFMs. Table S1: Lennard–Jones parameters of metal–organic frameworks (MOFs); Table S2: Lennard–Jones parameters and charges of adsorbates; Table S3: Optimal hyperparameters for SVM, KNN, DT, RF, and GBDT; Table S4: Optimal hyperparameters for LGBM and XGBoost; Table S5: Evaluation of seven ML algorithm for *P*; Table S6: Evaluation of seven ML algorithm for *S_perm_*: Table S7: Evaluation of XGBoost for *P*_CO_2__ and *P*_O_2__; Table S8: Evaluation of XGBoost for *P*_N_2__; Table S9: Evaluation of XGBoost for *S_perm_*_(CO_2_/O_2_)_ and *S_perm_*_(CO_2_/N_2_)_; Table S10: Seven top-performance MOFMs for CO_2_/N_2_/O_2_ separation [9,37,53,54,55,56,57,58,59,60,61].
