# Supplementary material for: Large-Scale Screening and Machine Learning for Metal–Organic Framework Membranes to Capture CO2 from Flue Gas
Source: Membranes (Basel). 2022 Jul 11;12(7):700. doi: 10.3390/membranes12070700 (PMC9321510; doi:10.3390/membranes12070700)
Supplement: Supplementary file 1 [file membranes-12-00700-s001.zip › membranes-1815179-supplementary.pdf]

Supplementary Material

Large-Scale Screening and Machine Learning for the Metal-Organic Framework Membranes to Capture CO_2 from Flue Gas

Yizhen Situ[1], Xueying Yuan[1], Xiangning Bai[1], Shuhua Li[1], Hong Liang[1], Xin Zhu[1]*, Bangfen Wang[1]* and Zhiwei Qiao[1,2]*

1 Guangzhou Key Laboratory for New Energy and Green Catalysis, School of Chemistry and Chemical Engineering, Guangzhou University, Guangzhou 510006, China

2 Joint Institute of Guangzhou University & Institute of Corrosion Science and Technology, Guangzhou University, Guangzhou 510006, China[*]

[*] Email: zqiao@gzhu.edu.cn

Table of contents

Lennard-Jones parameters of MOFs S2
Lennard-Jones parameters and charges of adsorbates S3
Univariate analysis S4
Machine learning S5
Evaluation of machine learning S13
References S19

Table S1. Lennard–Jones parameters of metal–organic frameworks (MOFs) [1].

Atom	ε/k_B [K]	σ [Å]	Atom	ε/k_B [K]	σ [Å]	Atom	ε/k_B [K]	σ [Å]
Ac	16.60	3.10	Ge	190.69	3.81	Po	163.52	4.20
Ag	18.11	2.80	Gd	4.53	3.00	Pr	5.03	3.21
Al	254.09	4.01	H	22.14	2.57	Pt	40.25	2.45
Am	7.04	3.01	Hf	36.23	2.80	Pu	8.05	3.05
Ar	93.08	3.45	Hg	193.71	2.41	Ra	203.27	3.28
As	155.47	3.77	Ho	3.52	3.04	Rb	20.13	3.67
At	142.89	4.23	I	170.57	4.01	Re	33.21	2.63
Au	19.62	2.93	In	301.39	3.98	Rh	26.67	2.61
B	90.57	3.64	Ir	36.73	2.53	Rn	124.78	4.25
Ba	183.15	3.30	K	17.61	3.40	Ru	28.18	2.64
Be	42.77	2.45	Kr	110.69	3.69	S	137.86	3.59
Bi	260.63	3.89	La	8.55	3.14	Sb	225.91	3.94
Bk	6.54	2.97	Li	12.58	2.18	Sc	9.56	2.94
Br	126.29	3.73	Lu	20.63	3.24	Se	146.42	3.75
C	52.83	3.43	Lr	5.53	2.88	Si	202.27	3.83
Ca	119.75	3.03	Md	5.53	2.92	Sm	4.03	3.14
Cd	114.72	2.54	Mg	55.85	2.69	Sn	285.28	3.91
Ce	6.54	3.17	Mn	6.54	2.64	Sr	118.24	3.24
Cf	6.54	2.95	Mo	28.18	2.72	Ta	40.75	2.82
Cl	114.21	3.52	N	34.72	3.26	Tb	3.52	3.07
Cm	6.54	2.96	Na	15.09	2.66	Tc	24.15	2.67
Co	7.04	2.56	Ne	21.13	2.66	Te	200.25	3.98
Cr	7.55	2.69	Nb	29.69	2.82	Th	13.08	3.03
Cu	2.52	3.11	Nd	5.03	3.18	Ti	8.55	2.83
Cs	22.64	4.02	No	5.53	2.89	TI	342.14	3.87
Dy	3.52	3.05	Ni	7.55	2.52	Tm	3.02	3.01
Eu	4.03	3.11	Np	9.56	3.05	U	11.07	3.02
Er	3.52	3.02	O	30.19	3.12	V	8.05	2.80
Es	6.04	2.94	Os	18.62	2.78	W	33.71	2.73
F	25.16	3.00	P	153.46	3.69	Xe	167.04	3.92
Fe	6.54	2.59	Pa	11.07	3.05	Y	36.23	2.98
Fm	6.04	2.93	Pb	333.59	3.83	Yb	114.72	2.99
Fr	25.16	4.37	Pd	24.15	2.58	Zn	62.39	2.46
Ga	208.81	3.90	Pm	4.53	3.16	Zr	34.72	2.78

Table S2. Lennard–Jones parameters and charges of adsorbates [2].

Atom	ε/k_B [K]	σ [Å]	Charge (e)	Atom	ε/k_B [K]	σ [Å]	Charge (e)
C_CO_2	27.0	2.80	+0.700	H_H_2S	50.0	2.50	+0.210
O_CO_2	79.0	3.05	–0.350	S_H_2S	122.0	3.60	0
CH_4	148.0	3.73	0	M_H_2S	0	0	–0.420
N_N_2	36.0	3.31	–0.482	H_H_2	0	0	+0.468
com_N_2	0	0	+0.964	com_H_2	36.7	2.96	–0.936
O_O_2	49.0	3.02	–0.113	He	10.9	2.64	0
com _O_2	0	0	+0.226				

Univariate analysis

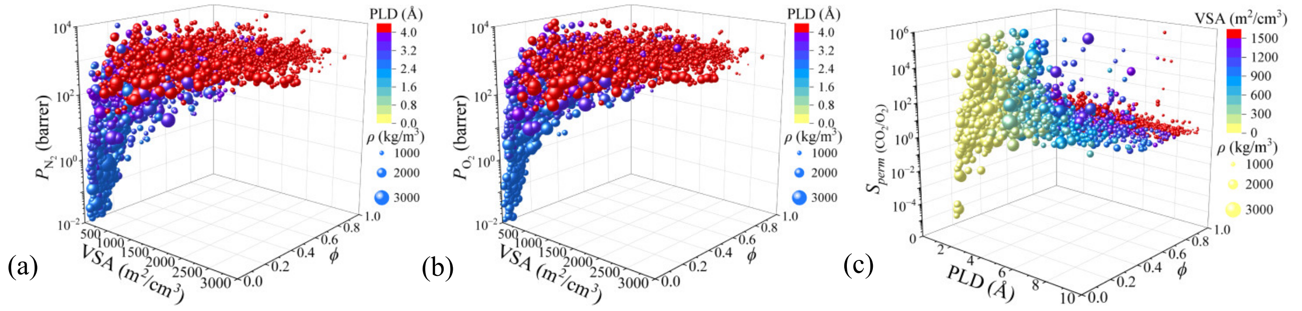

Figure S1. P_{N_2}/ P_{O_2}/$S_{perm\ (CO_2/O_2)}$ – PLD, ϕ, VSA, and ρ. In (a) and (b), the colors of ball represent the PLD of MOFMs and the sizes of ball represent ρ of MOFMs. In (c), the colors of ball represent the VSA of MOFMs and the sizes of ball represent ρ of MOFMs. (a) P_{N_2}; (b) P_{O_2}; (c) $S_{perm\ (CO_2/O_2)}$.

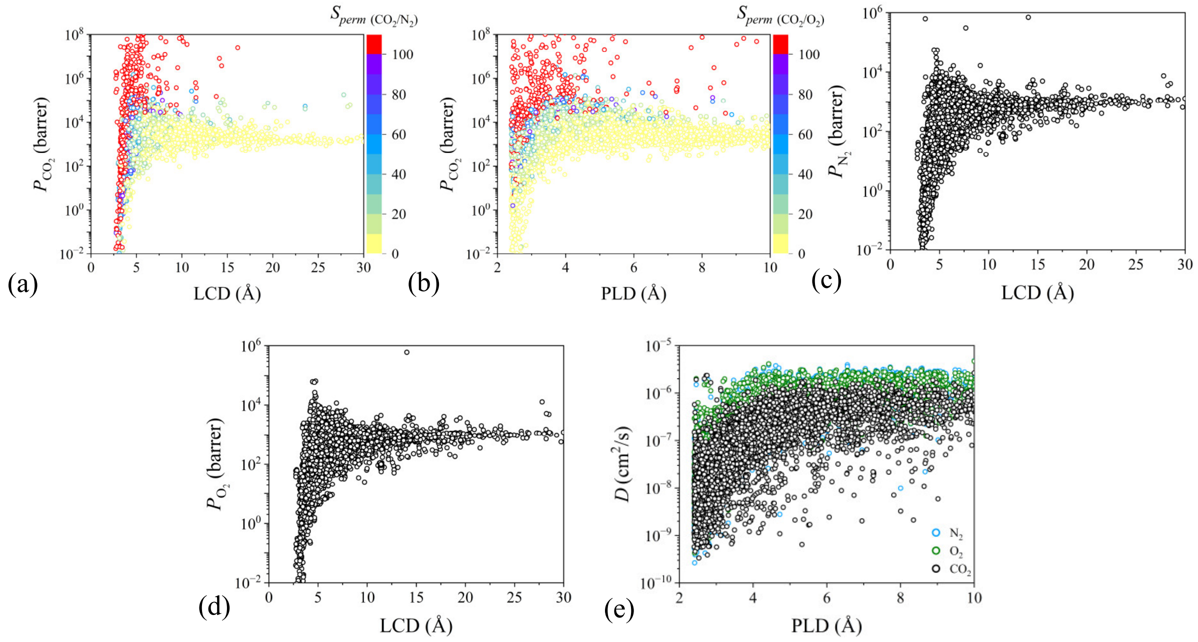

Figure S2. (a) LCD-P_{CO_2}-$S_{perm\ (CO_2/N_2)}$; (b) PLD-P_{CO_2}-$S_{perm\ (CO_2/O_2)}$; (c) P_{N_2}-LCD; (d) P_{O_2}-LCD; (e) D-PLD.

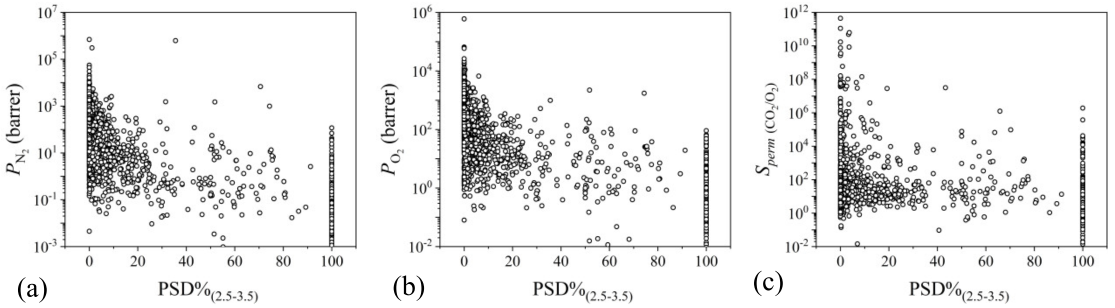

Figure S3. P /S_{perm} –PSD%$_{(2.5\text{-}3.5)}$. (a) P_{N_2}; (b)P_{O_2}; (c) $S_{perm\ (CO_2/O_2)}$.

Machine learning

In this work, for the analysis of the relative importance from six structural descriptors to the permeance performance of MOFMs, the prediction accuracy of seven classification ML algorithms[3] were compared, which are support vector machine, k-nearest neighbor, decision tree, random forest, gradient boosting decision trees, light gradient boosting machine, and extreme gradient boosting. In order to compare the relative importance to the P of different gas molecules and the S_{perm} of two binary gas pairs, a same calculation method for relative importance should be found. After simply comparing the prediction accuracy of above algorithms with 5-fold cross validation under the same data splitting state, XGBoost with optimal prediction accuracy was selected finally. However, because of different data characters, different prediction accuracy and stable performance may be gotten in different fold cross validation. Aiming to get a believable prediction result, the XGBoost model with k-fold cross validation (k=5, 10, and 15) was trained and tested in five times every time. In machine learning, 70% of data was used to train ML models and the else 30% of data was used to test the prediction accuracy of models. The prediction accuracy of models was evaluated by the accuracy (A), the sensitive (SEN), and the specificity (SPC), which were calculated by S (1)-S (3). In the following equation S (1)-S (3), TP represents true positives, TN represents true negatives, FP represents false positives, and FN represents false negatives, as shown in Fig.S3. Besides, the hyperparameters of above algorithms were found by BayesSearchCV.

$$\mathrm{A} = \frac{\mathrm{TP}+\mathrm{TN}}{\mathrm{TP}+\mathrm{TN}+\mathrm{FP}+\mathrm{FN}} \qquad \text{S (1)}$$

$$\mathrm{SEN} = \frac{\mathrm{TP}}{\mathrm{TP}+\mathrm{FN}} \qquad \text{S (2)}$$

$$\mathrm{SPC} = \frac{\mathrm{TN}}{\mathrm{TN}+\mathrm{FP}} \qquad \text{S (3)}$$

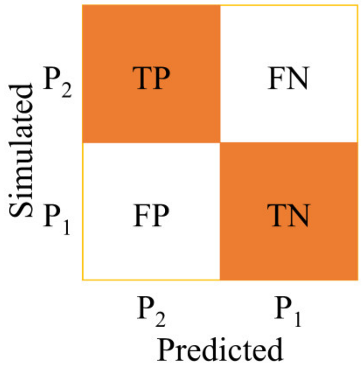

Figure S4. The calculation schematic diagram for the accuracy, sensitive, and specificity.

For extreme gradient boosting method, feature importance will be calculated after the boost tree is built. Normally, there are five methods for it to calculate the feature importance, which are based on 'weight', 'gain', 'cover', 'total_gain', and 'total_cover'. 'weight' is the number of times a feature is used to split the data across all trees. 'gain' is the average gain across all splits the feature is used in. 'cover' is the average coverage across all splits the feature is used in. 'total_gain' is the total gain across all splits the feature is used in. 'total_cover' is the total coverage across all splits the feature is used in. In this work, the method based on 'total_gain' was selected to calculate the feature importance.

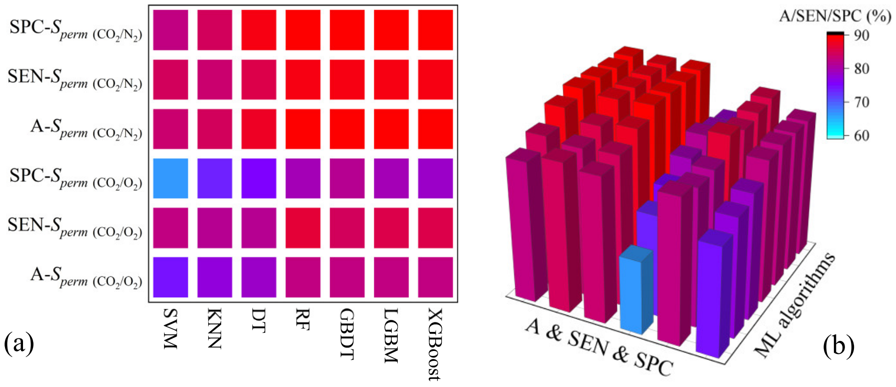

Figure S5. The accuracy, sensitive, and specificity comparison of seven algorithms for S_{perm} (CO_2/N_2) and S_{perm} (CO_2/O_2).

BayesSearchCV

BayesSearchCV[4] is an excellent method for hyperparameters searching, which tried out a fixed number of hyperparameters setting sampled from the specified distributions instead of all hyperparameter values. Besides, the hyperparameters are optimized by cross-validated search over hyperparameters settings in this process. In this work, the hyperparameters for BayesSearchCV follows the default setting except the fold number of cross validation.

***k*-fold cross validation**

Usually, the *k*-fold cross validation is used to model tuning to look for hyperparameters that make the model generalization performance optimal. Generally, the *k*-fold cross validation is to divide the data set into *k* parts in equal proportion, namely, the training set and the test set. The *k*-1 of folded data set are used to train model, while one of them is used for the validation of the resulting model. Finally, the parameter with the lowest mean error is selected for each parameter. The *k*-fold cross validation diagram is shown as follow.

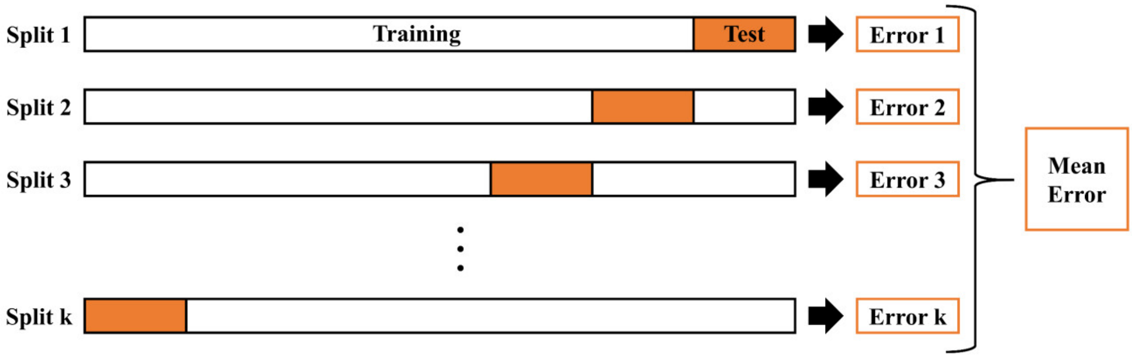

Figure S6. The diagram of *k*-fold cross validation.

k-nearest neighbor

k-nearest neighbors (KNN)[5] is a simple algorithm classifying objects based on a metric distance between testing features and each feature in the feature space. The classification results of objects samples belong to the classification of most of the k training set samples that are closest to the object sample.

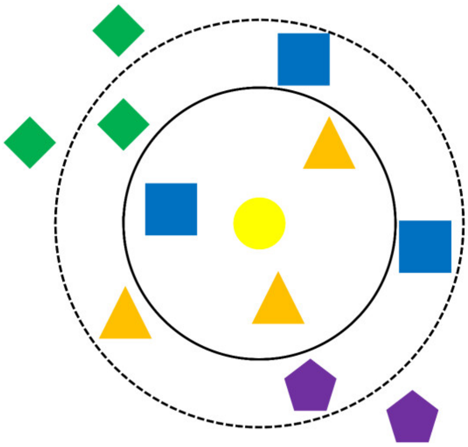

Figure S7. KNN algorithm model.

Support vector machine

As the statistical learning is its theoretical framework, support vector machine (SVM)[6] is a supervised learning algorithm, which regards the minimum deviation between the target value and the output value as its constraint condition rather than the target to realize. Looking for an optimal hyperplane in high-dimensional space and outputting the sample model, SVM introduces a tolerance constant ε (ε>0), as shown in Fig.S8. In this work, aiming to yield a better performance model, radial basis functions were introduced as kernel functions.

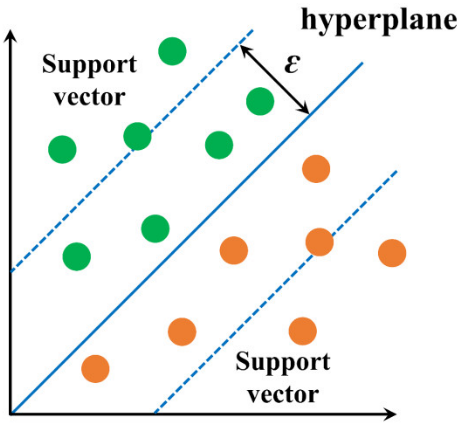

Figure S8. SVM algorithm model.

Decision tree

Decision tree (DT)[7] algorithm is a supervised learning algorithm used for both regression and classification analysis. For classification, DT model selects split features that maximizes the split-criterion gain over all possible splits of all features and choose the most important feature as the root node. Then, according to the splitting criterion, the binary splitting is carried out from root node to leaf node.

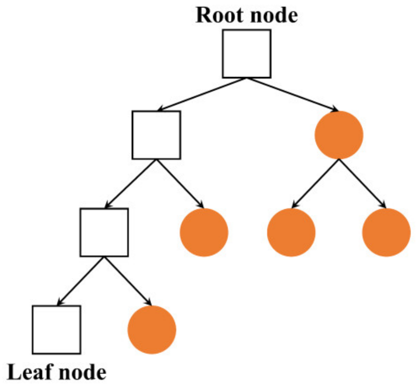

Figure S9. DT algorithm model.

Random forest

Random forest (RF)[8] algorithm is an algorithm based on DT algorithm, which randomly chooses N samples from X and randomly chooses K characters from all characters to build decision tree by the best separation characters which are tree nodes. Repeating above procedure, random forest is built by many decision trees and the classification with most votes over all trees is the final result of RF classification model.

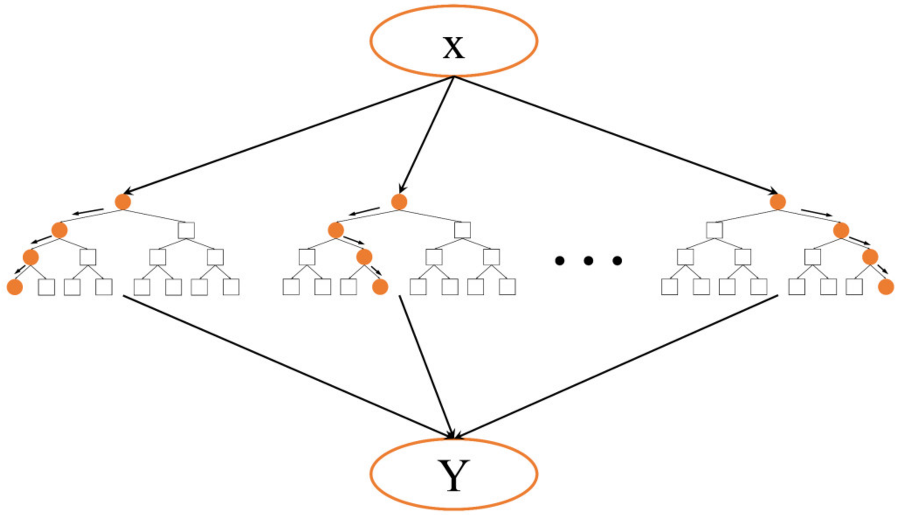

Figure S10. RF algorithm model.

Gradient boosting decision tree

As an ensemble method, aiming to provide a more accurate estimate of the response variable, the learning procedure of gradient boosting machine consecutively fits new models. There are three elements involving in gradient boosting, which are the loss function to be optimized, the weak learner to make predictions and the additive model to add weak learners to minimize the loss function. In this work, the decision tree is applied as the basic learner so that the algorithm is called gradient boosting decision tree (GBDT)[9]. The loss function we choose is the loss ‘deviance’, and the continuous error fitting is gotten in the learning process. The conclusions and residuals of all previous trees are learned and the current resident decision trees are fitted by each of decision trees.

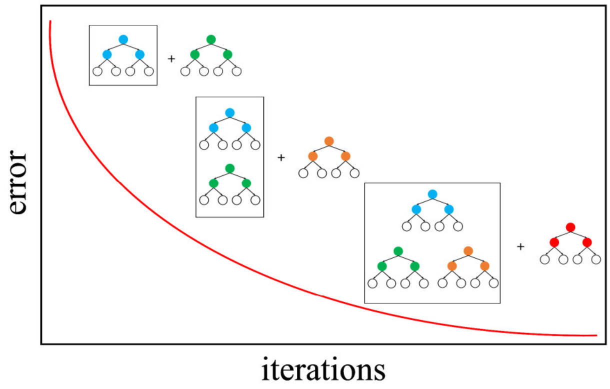

Figure S11. GBDT algorithm model.

Light gradient boosting machine

Light gradient boosting machine (LGBM)[10] is a histogram-based decision tree algorithm bucketing continuous feature values into discrete bins, which can speed up training and reduce memory usage. For categorical feature, instead of representing to one-hot encoding, LGBM splits them by partitioning into 2 subsets. Through sorting the categories according to the training objective at each split and sorting the histogram for a categorical feature according to its accumulated values, the best split on the sorted histogram was found. Besides, LGBM grows tree leaf-wise (best-first) instead of growing trees level-wise for the optimization in accuracy.

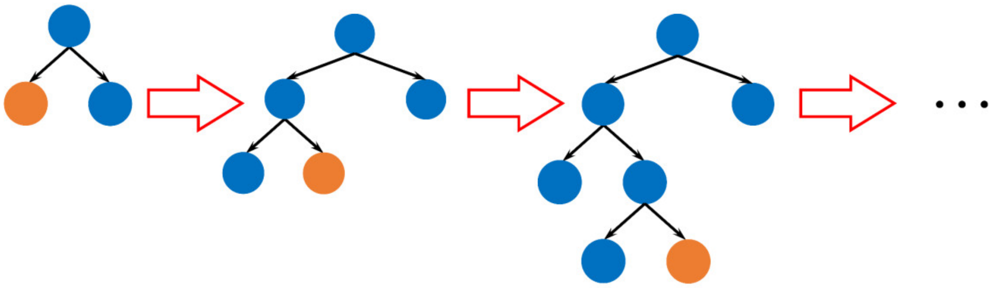

Figure S12. The leaf-wise tree growth schematic diagram of LGBM algorithm model;

Extreme gradient boosting

Extreme gradient boosting (XGBoost)[11] is an optimized machine learning system with the pursuance of highest speed and efficiency, which is essentially a gradient boosting decision tree. The core of XGBoost is an ensemble algorithm based on a gradient boosting tree, in which there are three parts: the ensemble algorithm itself, the weak learner and other processes in the application. When trees are added, the gradient descent procedure is applied to minimize the loss. Actually, above process is to learn new function to fit the residuals predicted last time until the satisfactory effect is achieved. After k trees are gotten from training, the sample scores are need to predict. Based on the sample characters, each leaf node will fall into a corresponding leaf node, and each leaf node will correspond to a score. Finally, the prediction score is the sum of scores predicted by each tree. Additionally, with the rest of same operations in the gradient boosting tree algorithm, the regular terms are added into the objective function based on the original loss function by XGBoost, and the loss function is taken place by the second-order Taylor approximation, which can extremely optimize the objective function.

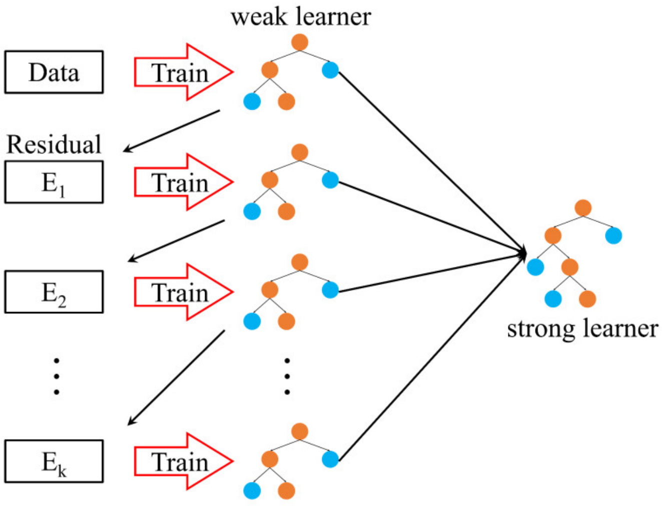

Figure S13. XGBoost algorithm model.

Optimal hyperparameters for algorithms

Table S3. Optimal hyperparameters for SVM, KNN, DT, RF, and GBDT.

Algorithm	Hyperparameters	P_{CO_2}	P_{O_2}	P_{N_2}	S_{perm} (CO_2/O_2)	$S_{perm}(CO_2/N_2)$
SVM	C	500	495	495	1	204
	kernel			rbf		
	degree	1	496	3	500	1
	gamma			scale		
	tol			1×10^{-4}		
KNN	n_neighbors	42	13	49	48	39
	weights			distance		
	algorithm			auto		
	leaf_size	42	50	45	42	50
DT	criterion			gini		
	splitter			best		
	max_depth	11	15	8	8	7
	min_samples_split	20	13	2	20	2
	min_samples_leaf	2	15	2	20	2
RF	criterion			gini		
	n_estimators	426	205	461	302	334
	max_depth	14	13	19	15	18
	min_samples_split	11	17	17	6	6
	min_samples_leaf	4	5	2	2	2
GBDT	loss			deviance		
	learning_rate	0.2625	0.1772	0.4045	0.2514	0.8432
	n_estimators	438	326	426	100	483
	min_samples_split	5	10	17	2	20
	min_samples_leaf	2	15	2	2	3
	max_depth	19	8	11	None	12
	subsample			1.0		
	criterion			friedman_mse		

Table S4. Optimal hyperparameters for LGBM and XGBoost.

Algorithm	Hyperparameters	P_{CO_2}	P_{O_2}	P_{N_2}	S_{perm} (CO_2/O_2)	S_{perm} (CO_2/N_2)
LGBM	boosting_type			gbdt		
	colsample_bytree	0.8935	0.7501	0.8440	0.7998	0.8372
	learning_rate	0.0754	0.1	0.0963	0.0225	0.1
	max_bin	300	100	207	260	250
	max_depth	11	8	15	20	20
	min_child_samples	1.0	18	1	2	4
	min_child_weight	0.1723	0.0010	0.4339	0.2832	0.0612
	min_split_gain	0.0703	0.0	0.0	0.0283	0.0
	n_estimators	419	300	300	390	500
	num_leaves	500	438	500	374	10
	reg_alpha	0.2703	0.2859	1.0	0.2972	0.9320
	reg_lambda	1.0	0.4895	0.4484	0.7179	0.2324
	subsample	0.9576	0.5745	0.5	0.9336	0.8849
	subsample_freq	9	74	40	49	0
	subsample_for_bin			200000		
	importance_type			gain		
XGBoost	objective			binary:logistic		
	booster			gbtree		
	colsample_bytree	1.0	1.0	1.0	1.0	0.8690
	gamma	0.0717	0.0965	0	0.1	0.0016
	learning_rate	0.0495	0.0379	0.0977	0.1062	0.1263
	max_depth	20	14	20	19	9
	min_child_weight	0.5562	0.1797	0.1	0.7442	0.3900
	n_estimators	303	193	100	274	271
	reg_alpha	0.6775	0.2605	1	0.6773	0.1098
	reg_lambda	0.8886	0.2534	0.1	0.8078	0.9008
	subsample	0.7748	0.5	0.5	0.5357	0.6700
	importance_type			total_gain		
	nthread			4		

Evaluation of machine learning

Table S5. Evaluation of seven ML algorithm for *P*.

	Algorithm	Training set			Test set		
		Accuracy	Specificity	Sensitive	Accuracy	Specificity	Sensitive
P_{CO_2}	SVM	0.69	0.54	0.84	0.69	0.54	0.83
	KNN	1.00	1.00	1.00	0.72	0.67	0.76
	DT	0.86	0.88	0.85	0.76	0.77	0.75
	RF	0.92	0.90	0.93	0.80	0.78	0.82
	GBDT	1.00	1.00	1.00	0.82	0.80	0.83
	LGBM	1.00	1.00	1.00	0.81	0.79	0.83
	XGBoost	0.98	0.98	0.99	0.81	0.79	0.83
P_{O_2}	SVM	0.78	0.77	0.80	0.81	0.80	0.82
	KNN	1.00	1.00	1.00	0.80	0.80	0.80
	DT	0.90	0.90	0.89	0.87	0.89	0.86
	RF	0.93	0.92	0.94	0.90	0.89	0.92
	GBDT	1.00	1.00	1.00	0.89	0.88	0.90
	LGBM	0.98	0.97	0.98	0.89	0.88	0.90
	XGBoost	1.00	1.00	1.00	0.90	0.89	0.91
P_{N_2}	SVM	0.80	0.81	0.80	0.82	0.82	0.82
	KNN	1.00	1.00	1.00	0.82	0.81	0.83
	DT	0.92	0.89	0.96	0.86	0.80	0.91
	RF	0.95	0.94	0.96	0.90	0.88	0.92
	GBDT	1.00	1.00	1.00	0.90	0.87	0.92
	LGBM	0.99	0.99	0.99	0.89	0.88	0.90
	XGBoost	1.00	1.00	1.00	0.90	0.88	0.91

Table S6. Evaluation of seven ML algorithm for S_{perm}.

	Algorithm	Training set			Test set		
		Accuracy	Specificity	Sensitive	Accuracy	Specificity	Sensitive
$S_{perm\ (CO_2/O_2)}$	SVM	0.76	0.69	0.84	0.75	0.67	0.83
	KNN	1.00	1.00	1.00	0.77	0.73	0.81
	DT	0.83	0.81	0.86	0.79	0.76	0.82
	RF	0.95	0.94	0.97	0.83	0.79	0.86
	GBDT	1.00	1.00	1.00	0.83	0.81	0.84
	LGBM	1.00	1.00	1.00	0.83	0.80	0.85
	XGBoost	0.97	0.97	0.98	0.82	0.79	0.86
$S_{perm\ (CO_2/N_2)}$	SVM	0.83	0.82	0.84	0.83	0.82	0.84
	KNN	1.00	1.00	1.00	0.84	0.85	0.84
	DT	0.90	0.91	0.88	0.87	0.88	0.86
	RF	0.97	0.97	0.96	0.89	0.90	0.89
	GBDT	1.00	1.00	1.00	0.89	0.90	0.89
	LGBM	0.97	0.97	0.96	0.89	0.89	0.89
	XGBoost	0.97	0.97	0.97	0.89	0.89	0.89

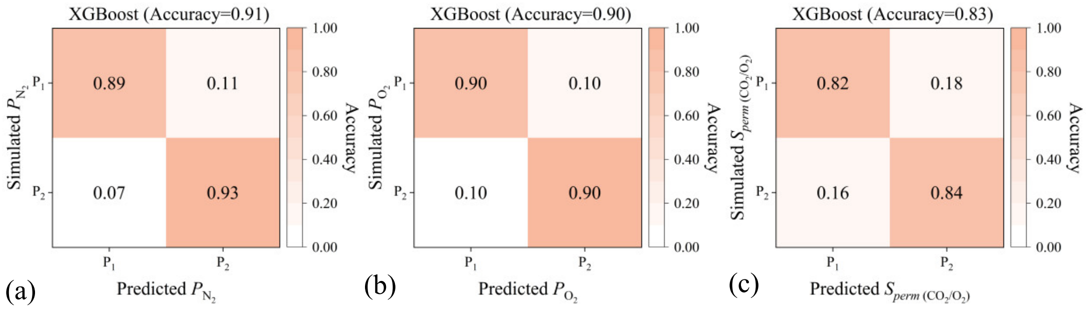

Figure S14. Confusion matrix from best model. (a)P_{N_2}; (b) P_{O_2}; (c) $S_{perm\ (CO_2/O_2)}$.

Table S7. Evaluation of XGBoost for P_{CO_2} and P_{O_2}.

			Training set			Test set		
			Accuracy	Specificity	Sensitive	Accuracy	Specificity	Sensitive
P_{CO_2}	5-fold cross validation	1	1.00	1.00	1.00	0.80	0.80	0.80
		2	0.96	0.95	0.97	0.80	0.81	0.80
		3	0.99	0.99	1.00	0.81	0.81	0.81
		4	1.00	1.00	1.00	0.81	0.80	0.81
		5	1.00	1.00	1.00	0.82	0.80	0.83
		Average	0.99	0.99	0.99	0.81	0.80	0.81
	10-fold cross validation	1	0.98	0.97	0.99	0.80	0.80	0.81
		2	1.00	1.00	1.00	0.81	0.81	0.80
		3	1.00	1.00	1.00	0.81	0.81	0.81
		4	1.00	1.00	1.00	0.81	0.81	0.82
		5	1.00	1.00	1.00	0.81	0.80	0.81
		Average	1.00	0.99	1.00	0.81	0.81	0.81
	15-fold cross validation	1	1.00	1.00	1.00	0.79	0.80	0.79
		2	1.00	1.00	1.00	0.82	0.82	0.83
		3	1.00	1.00	1.00	0.80	0.80	0.80
		4	0.99	0.98	0.99	0.80	0.78	0.82
		5	1.00	1.00	1.00	0.82	0.83	0.80
		Average	1.00	1.00	1.00	0.81	0.80	0.81
P_{O_2}	5-fold cross validation	1	1.00	1.00	1.00	0.88	0.89	0.88
		2	1.00	1.00	1.00	0.88	0.88	0.89
		3	1.00	1.00	1.00	0.90	0.90	0.90
		4	1.00	1.00	1.00	0.88	0.87	0.88
		5	1.00	1.00	1.00	0.88	0.88	0.88
		Average	1.00	1.00	1.00	0.88	0.88	0.89
	10-fold cross validation	1	1.00	1.00	1.00	0.88	0.88	0.87
		2	0.99	0.99	0.99	0.89	0.87	0.90
		3	1.00	1.00	1.00	0.89	0.87	0.91
		4	0.96	0.95	0.96	0.89	0.89	0.89
		5	1.00	1.00	1.00	0.88	0.90	0.87
		Average	0.99	0.99	0.99	0.89	0.88	0.89
	15-fold cross validation	1	1.00	0.99	1.00	0.90	0.91	0.89
		2	1.00	1.00	1.00	0.89	0.88	0.90
		3	1.00	1.00	1.00	0.88	0.90	0.86
		4	1.00	1.00	1.00	0.90	0.90	0.90
		5	0.99	0.98	0.99	0.90	0.89	0.91
		Average	1.00	0.99	1.00	0.89	0.89	0.89

Table S8. Evaluation of XGBoost for P_{N_2}.

		Training set			Test set		
		Accuracy	Specificity	Sensitive	Accuracy	Specificity	Sensitive
5-fold cross validation	1	1.00	1.00	1.00	0.89	0.87	0.90
	2	1.00	1.00	1.00	0.90	0.88	0.91
	3	1.00	0.99	1.00	0.90	0.88	0.91
	4	1.00	1.00	1.00	0.89	0.87	0.91
	5	0.99	0.98	0.99	0.89	0.85	0.92
	Average	1.00	1.00	1.00	0.89	0.87	0.91
10-fold cross validation	1	1.00	1.00	1.00	0.89	0.87	0.90
	2	1.00	1.00	1.00	0.90	0.89	0.90
	3	1.00	1.00	1.00	0.90	0.88	0.91
	4	1.00	1.00	1.00	0.90	0.91	0.90
	5	1.00	1.00	1.00	0.90	0.89	0.91
	Average	1.00	1.00	1.00	0.90	0.89	0.90
15-fold cross validation	1	1.00	1.00	1.00	0.90	0.90	0.90
	2	1.00	1.00	1.00	0.90	0.90	0.90
	3	0.99	0.98	0.99	0.89	0.87	0.90
	4	1.00	1.00	1.00	0.88	0.86	0.90
	5	0.99	0.99	1.00	0.91	0.89	0.93
	Average	1.00	1.00	1.00	0.89	0.88	0.91

Table S9. Evaluation of XGBoost for $S_{perm\ (CO_2/O_2)}$ and $S_{perm\ (CO_2/N_2)}$.

			Training set			Test set		
			Accuracy	Specificity	Sensitive	Accuracy	Specificity	Sensitive
$S_{perm\ (CO_2/O_2)}$	5-fold cross validation	1	1.00	1.00	1.00	0.81	0.79	0.84
		2	1.00	1.00	1.00	0.81	0.78	0.85
		3	0.98	0.98	0.99	0.83	0.81	0.85
		4	1.00	1.00	1.00	0.83	0.81	0.85
		5	1.00	1.00	1.00	0.82	0.80	0.85
		Average	1.00	0.99	1.00	0.82	0.80	0.85
	10-fold cross validation	1	0.98	0.97	0.99	0.81	0.78	0.84
		2	1.00	1.00	1.00	0.82	0.79	0.86
		3	1.00	1.00	1.00	0.83	0.81	0.84
		4	1.00	1.00	1.00	0.83	0.82	0.84
		5	1.00	1.00	1.00	0.83	0.81	0.85
		Average	0.99	0.99	1.00	0.82	0.80	0.84
	15-fold cross validation	1	1.00	1.00	1.00	0.82	0.79	0.85
		2	0.99	0.99	0.99	0.83	0.79	0.87
		3	1.00	1.00	1.00	0.83	0.79	0.86
		4	0.99	0.99	0.99	0.82	0.77	0.87
		5	1.00	1.00	1.00	0.82	0.79	0.85
		Average	1.00	1.00	1.00	0.82	0.79	0.86
$S_{perm\ (CO_2/N_2)}$	5-fold cross validation	1	0.97	0.97	0.96	0.87	0.88	0.86
		2	1.00	1.00	1.00	0.88	0.87	0.89
		3	0.99	0.99	0.99	0.88	0.87	0.88
		4	1.00	1.00	1.00	0.89	0.88	0.90
		5	1.00	1.00	1.00	0.88	0.88	0.89
		Average	0.99	0.99	0.99	0.88	0.88	0.89
	10-fold cross validation	1	0.99	0.99	0.99	0.88	0.87	0.88
		2	1.00	1.00	1.00	0.89	0.89	0.89
		3	1.00	1.00	1.00	0.87	0.87	0.86
		4	1.00	1.00	1.00	0.88	0.88	0.88
		5	1.00	1.00	1.00	0.89	0.89	0.88
		Average	1.00	1.00	1.00	0.88	0.88	0.88
	15-fold cross validation	1	1.00	1.00	1.00	0.88	0.87	0.89
		2	1.00	1.00	1.00	0.89	0.90	0.88
		3	0.97	0.97	0.97	0.88	0.88	0.88
		4	1.00	1.00	1.00	0.88	0.89	0.88
		5	0.99	0.99	0.99	0.87	0.87	0.87
		Average	0.99	0.99	0.99	0.88	0.88	0.88

The top-performance MOFMs for $CO_2/N_2/O_2$ separation

Table S10 Seven top-performance MOFMs for $CO_2/N_2/O_2$ separation.

CSD	LCD (Å)	ϕ	VSA (m^2/cm^3)	PLD (Å)	ρ (kg/m^3)	PSD%$_{(2.5\text{-}3.5)}$	P (barrer)			S_{perm}		
							CO_2	O_2	N_2	CO_2/O_2	CO_2/N_2	$CO_2/(N_2+O_2)$
CARGEI	4.11	0.09	130.06	3.66	3443.32	0.00	2.98×10^{7}	21.89	25.09	1.36×10^{6}	1.19×10^{6}	1.27×10^{6}
YUJWAD	3.88	0.26	16.62	2.64	1429.41	3.35	1.02×10^{12}	20.87	62.36	4.89×10^{10}	1.64×10^{10}	2.45×10^{10}
YUJWOR*	3.84	0.26	10.46	2.71	1414.41	3.59	1.73×10^{11}	20.48	52.67	8.43×10^{9}	3.28×10^{9}	4.72×10^{9}
YUJWUX*	3.88	0.27	23.78	2.69	1411.36	2.03	5.65×10^{8}	20.77	97.19	2.72×10^{7}	5.82×10^{6}	9.58×10^{6}
RIPWEU	5.37	0.24	591.19	3.29	3958.43	3.08	4.93×10^{9}	47.78	91.17	1.03×10^{8}	5.41×10^{7}	7.10×10^{7}
VEHNED	3.81	0.23	134.63	2.56	1435.20	2.58	6.25×10^{8}	16.02	59.76	3.90×10^{7}	1.05×10^{7}	1.65×10^{7}
WOCJII	4.01	0.13	5.53	2.57	1574.83	12.15	1.88×10^{7}	4.85	5.69	3.88×10^{6}	3.30×10^{6}	3.57×10^{6}

* Additional Database Identifiers in CCDC

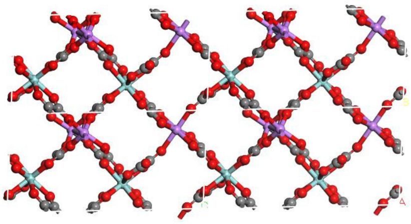

(a)

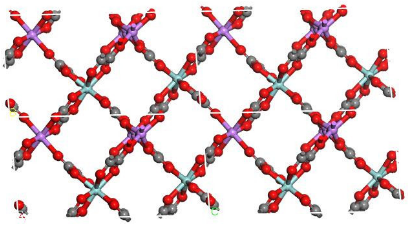

(b)

Figure.S15 Atomistic structures of top-performance MOFMs.
(a) YUJWOR; (b) YUJWUX;

References

1 Rappe, A. K.; Casewit, C. J.; Colwell, K. S.; Goddard, W. A.; Skiff, W. M. UFF A Full Periodic Table Force Field for Molecular Mechanics and Molecular Dynamics Simulation. *J. Am. Chem. Soc.* **1992**, *114* (25), 10024-10035.

2 Shah, M. S.; Tsapatsis, M.; Siepmann, J. I. Development of the Transferable Potentials for Phase Equilibria Model for Hydrogen Sulfide. *J. Phys. Chem. B* **2015**, *119* (23), 7041-7052. DOI: 10.1021/acs.jpcb.5b02536.

3 Fabian Pedregosa; Varoquaux, G.; Gramfort, A.; Michel, V.; Thirion, B.; Grisel, O.; Blondel, M.; Prettenhofer, P.; Weiss, R.; Dubourg, V.; et al. Scikit-learn: Machine Learning in Python. *J. Mach. Learn. Res.* **2011**, *12* (85), 2825-2830.

4 http://scikit-optimize.github.io/stable/modules/bayessearchcv.html.

5 COVER, T. M.; HART, P. E. Nearest Neighbor Pattern Classification *IEEE Trans. Inf Theory* **1967**, *13* (1), 21.

6 Scholkopf, B.; Sung, K.-K.; Burges, C. J. C.; Girosi, F.; Niyogi, P.; Poggio, T.; Vapnik, V.; Tran, I. Comparing Support Vector Machines with Gaussian Kernels to Radial Basis Function Classiers. *IEEE Trans. Signal Process.* **1997**, *45*, 2758-2765.

7 Qiao, Z.; Li, L.; Li, S.; Liang, H.; Zhou, J.; Snurr, R. Q. Molecular fingerprint and machine learning to accelerate design of high-performance homochiral metal–organic frameworks. *AlChE J.* **2021**, *67* (10), e17352. DOI: 10.1002/aic.17352.

8 Yin, F.; Shao, X.; Zhao, L.; Li, X.; Zhou, J.; Cheng, Y.; He, X.; Lei, S.; Li, J.; Wang, J. Predicting prognosis of endometrioid endometrial adenocarcinoma on the basis of gene expression and clinical features using Random Forest. *Oncol. Lett.* **2019**, *18* (2), 1597-1606. DOI: 10.3892/ol.2019.10504.

9 Friedman, J. H. Greedy function approximation: A gradient boosting machine. *Ann. Stat.* **2001**, *29* (5), 1189-1232.

10 https://lightgbm.readthedocs.io/en/latest/Features.html.

11 https://xgboost.readthedocs.io/en/stable/.
